# Hydrophobe Containing Polypeptoids Complex with Lipids and Induce Fusogenesis of Lipid Vesicles

**DOI:** 10.1021/acs.jpcb.0c11477

**Published:** 2021-03-17

**Authors:** Marzhana Omarova, Yueheng Zhang, Igor Kevin Mkam Tsengam, Jibao He, Tianyi Yu, Donghui Zhang, Vijay John

**Affiliations:** † Department of Chemical and Biomolecular Engineering, Tulane University, New Orleans, Louisiana 70118, United States; ‡ Department of Chemistry, Louisiana State University, Baton Rouge, Louisiana 70803, United States; § Coordinated Instrumentation Facility, Tulane University, New Orleans, Louisiana 70118, United States

## Abstract

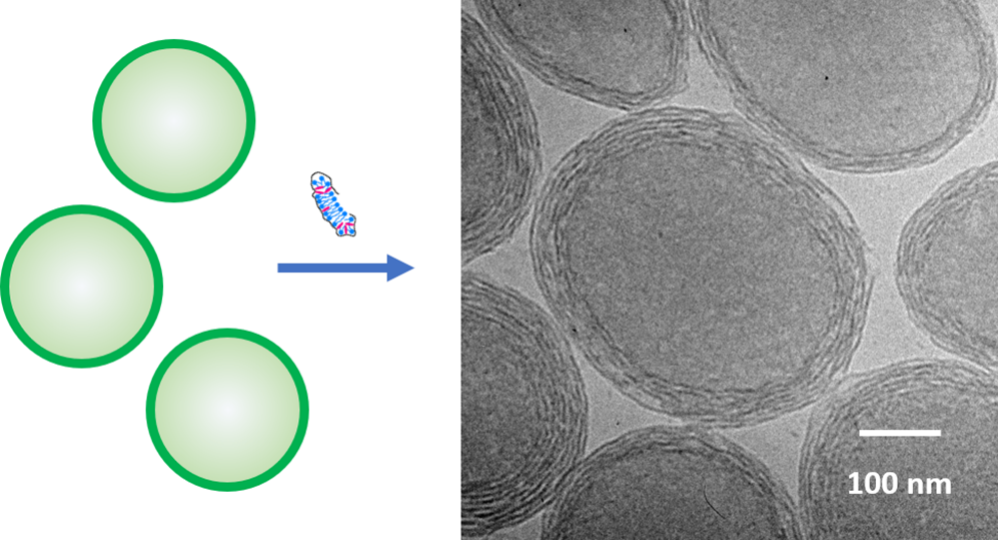

The hydrophobic effect of alkyl group insertion into phospholipid bilayers is exploited in modifying and modulating vesicle structure. We show that amphiphilic polypeptoids (peptide mimics) with *n*-decyl side chains, which we term as hydrophobe-containing polypeptoids (HCPs), can insert the alkyl hydrophobes into the membrane bilayer of phospholipid-based vesicles. Such insertion leads to disruption of the liposomes and the formation of HCP–lipid complexes that are colloidally stable in aqueous solution. Interestingly, when these complexes are added to fresh liposomes, remnant uncomplexed hydrophobes (the *n*-decyl groups) bridge liposomes and fuse them. The fusion leads to the engulfing of liposomes and the formation of multilayered vesicles. The morphology of the liposome system can be changed from stopping fusion and forming clustered vesicles to the continued formation of multilayered liposomes simply by controlling the amount of the HCP–lipid complex added. The entire procedure occurs in aqueous systems without the addition of any other solvents. There are several implications to these observations including the biological relevance of mimicking fusogenic proteins such as the SNARE proteins and the development of new drug delivery technologies to impact delivery to cell organelles.

## Introduction

Synthetic lipid vesicles or liposomes are a useful and convenient platform for research on model cell membrane ever since their first discovery by Bangham et al.^1^ with the self-assembly of such closed systems having implications to understanding the origin of life.^2,3^ Liposomes offer a number of advantages in drug delivery applications, such as a simple and scalable method of preparation and designability suitable for encapsulation of small molecules^4^ and nucleic acids.^5^ A number of drug formulations use liposomes or lipid-based nanoparticles in the clinical trials currently^5,6^ with a few approved for medical use in drug delivery.^7,8^

The mechanistic understanding of the formation and transformation of single bilayer-based vesicles to multilamellar vesicles is a continuing area of active research with implications to the fundamental knowledge of biological systems and to applied aspects of drug delivery. Multilamellar lipid vesicles formed through high-energy shear are well documented,^9,10^ but the formation of such multilamellar vesicles is imprecise; it is likely that fragile biomolecules in the vesicles are degraded through such shear effects. Recent interesting work describes the use of dendrimersomes or multilamellar vesicles formed using amphiphilic Janus dendrimers by injecting THF solutions of the dendrimer into water or a buffer.^11,12^ These are multistep synthetic processes involving the use of organic solvents, and their nontoxicity remains to be determined. Other recent examples include the use of rodlike oligofluorenes in a acetonitrile–water mixed solvent.^13^

Our work is based on a specific manifestation of the hydrophobic effect where alkyl hydrophobes on the backbone of a water-soluble biopolymer insert into membrane lipid bilayers. The concepts of such insertion are well established and are the reason detergents lyse cell membranes. In the specific system studied here, the biopolymer is a polypeptoid. Peptoids are a class of peptide mimics where the substituents are on the nitrogen rather than the carbon atoms.^14,15^ These polymers are therefore structurally similar to peptides.^16,17^ However, they are more resistant against degradation by proteases due to their N-substitution at the amide bond which provides a steric hindrance to the action of proteases.^18−21^ The biodegradabilty of peptoids renders them useful in biomedical applications.^22−24^

**Figure 1:**
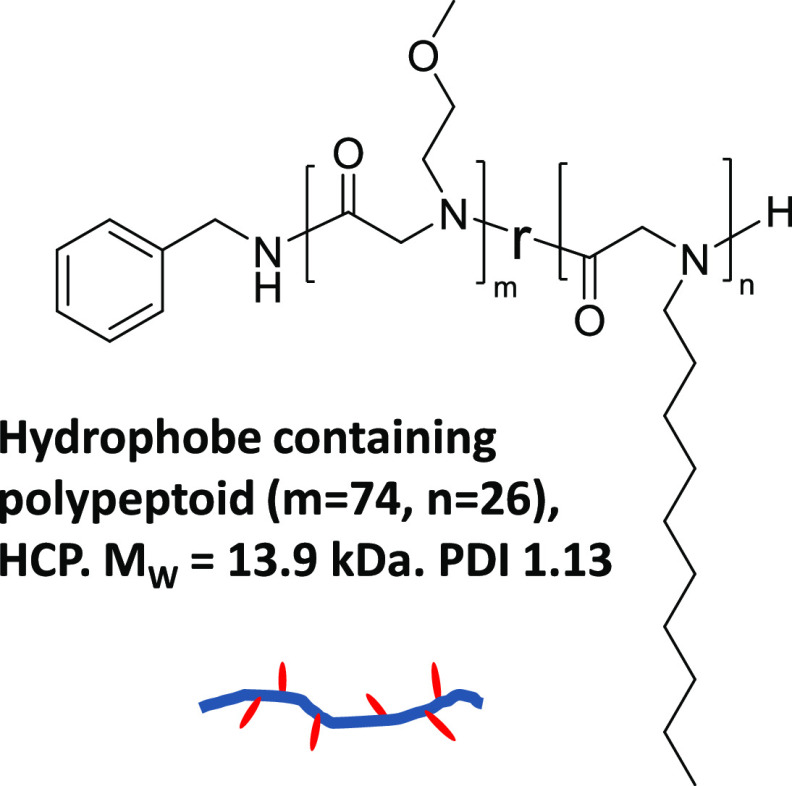
Molecular weight and structure of hydrophobe containing polypeptoid (HCP).

We specifically focus on the use of a hydrophobe containing polypeptoid (HCP) with ∼100 monomer units with a random placement of about 25% of the *N*-2-methoxyethyl group substituted by *n*-decyl groups (C10) which form the hydrophobes attached to the backbone of the water-soluble polymer, thus conferring a degree of amphiphilicity to the polymer. The detailed synthesis of the polymer is reported in our earlier publications (and additionally summarized in the materials and methods section).^25^Figure 1 illustrates the structure of the polymer.

**Figure 2:**
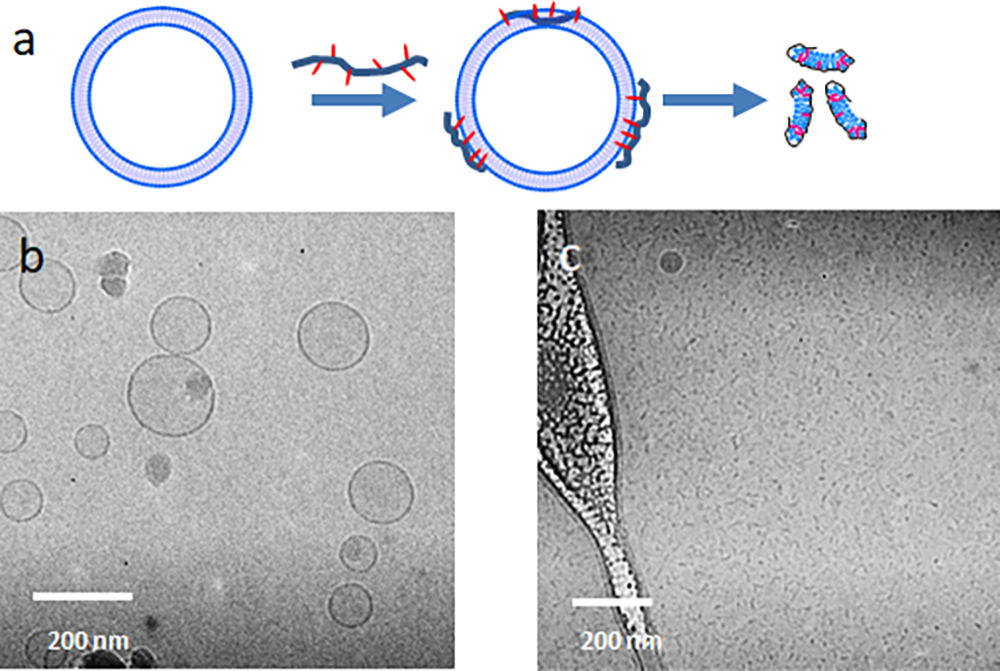
(a) Schematic to describe liposome disruption by HCP. (b) Typical cryo-TEM of unilamellar liposomes used in this study. (c) Cryo-TEM of the HCP–lipid complex formed by the addition of 0.5 wt % HCP to liposomes containing 0.25 wt % lipid (the dark region on the left is a part of lacey carbon substrate).

In our earlier work,^25,26^ we found that adding small amounts of HCP to phosphatidylcholine-based liposomes led to disruption of the liposomes with the observation of the formation of two- and three-layered liposomes. With sufficient addition of HCP at a composition of 0.25 wt % lipid to 0.5 wt % HCP, all liposomes become fragmented with the formation of HCP–lipid complexes. Figure 2 illustrates the concept and supports our previous finding^25,26^ that such complexes are formed upon the disruption of liposomes through the addition of HCPs from liposomes with a diameter on the order of 100 nm. Figure 2a is a schematic of the mechanism of hydrophobe insertion into the liposome and the resulting disruption into HCP–lipid complexes with an approximate 9:1 lipid:HCP molar ratio. We posit that the localized insertion of hydrophobes damages the membrane integrity leading to the disruption of the liposome, inducing transition from liposomes (Figure 2b) to HCP–lipid complexes (Figure 2c). These nanoscale HCP–lipid complexes were also characterized by small-angle neutron scattering (SANS) in addition to cryo-TEM (Figure 2c) to indicate elongated small wormlike entities with a 5.1 nm radius of gyration, a 2 nm radius, and a 38 nm contour length as calculated from the flexible cylinder model fitting.^25^ We have also shown that such HCP–lipid complexes can sustain hydrophobic drug moieties and are easily able to enter mammalian cells, leading to potential applications in drug delivery systems.^26^

The focus of this paper is on a second manifestation of the concept of hydrophobe insertion into lipid bilayers that is equally interesting with the potential to further fundamental understanding of the hydrophobic effect and lead to new applications. The work builds on our previous findings of layered vesicle formation and shows that HCP–lipid complexes can induce fusogenesis of unilamellar vesicles to multilayered vesicles. We advance mechanistic hypotheses to explain these fusogenic properties based on experimental results obtained through optical and cryogenic transmission electron microscopy. The concept relates to the addition of the HCP–lipid complex to new liposomes. In this instance, all hydrophobes of the HCP do not have the ability to insert into the liposome bilayer as they interact with lipids in the complex. We have found that these HCP–lipid complexes are therefore unable to disrupt the liposomes. Rather, they remodel liposomes to build multilayered vesicles. The transition is caused by the amphiphilic polypeptoid which acts as the main driver of reorganization in the lipid containing system. We also show that the formation of these multilayered liposomes is the result of the fusogenic properties of the HCP–lipid complexes, which can also arrest intermediate structures, opening up possibilities to further modulating vesicle structure. The details of these finding are described in the following sections of the paper.

## Experimental Methods

### Materials

l-α-Phosphatidylcholine (PC, >95%, from soy) was purchased from Avanti Polar Lipids. Fluorescein isothiocyanate–dextran (FITC–dextran, *M*_w_ 3–5 kDa) was purchased from Sigma-Aldrich. All other chemicals and solvents were purchased from Sigma-Aldrich and used as received unless otherwise noted. The solvents used for polymerization were further purified by using alumina columns under argon protection. CD_2_Cl_2_ and CDCl_3_ were purchased from Cambridge Isotope laboratories. ^1^H NMR was collected by Bruker AV-400 III spectrometer at 298 K and analyzed by using Topspin software. Chemical shifts (δ) given in parts per million (ppm) were referenced to protio impurities.

### Hydrophobe Containing Polypeptoid (HCP) Synthesis

*n*-Decylglycine derived *N*-carboxyanhydride (De-NCA) and *N*-methoxyethylglycine derived *N-*carboxyanhydride (MeOEt*-*NCA) monomers were synthesized by a published procedure.^27^ The HCPs were synthesized through primary amine-initiated ring-opening polymerization of the corresponding R-NCA monomers as reported previously.^25^ Copolymerization of *N*-methoxyethyl NCA and *n*-decyl NCA yields a random copolymer of *N*-methoxyethyl glycine units and *n*-decylglycine units. In a typical synthesis, in a glovebox, stock solutions of MeOEt-NCA (1.3 mL, 0.52 mmol, 0.4 M) and De-NCA (433 μL, 0.17 mmol, 0.4 M) in THF were premixed before the addition of benzylamine stock solution (74.8 μL, 6.9 μmol, 92.7 mM) in THF. The mixture was stirred at 50 °C under a nitrogen atmosphere for 72 h to reach complete conversion. The polymerization conversion was tracked by monitoring the disappearance of the −C=O peak at 1780 and 1740 cm^–1^ in the reaction aliquots taken over time by using FT-IR spectroscopy. The volatiles were removed under vacuum by using a Schlenk line. The crude polymer was further purified by redissolved in DCM and precipitated with ample hexanes twice to obtain the final product as a white solid (61.6 mg, 65.6% yield). The polymer composition was determined by end-group analysis using ^1^H NMR, and the polymer polydispersity index (PDI) was obtained by using size-exclusion chromatography (SEC).

### Size-Exclusion Chromatography (SEC)

SEC experiments were performed in DMF with 0.1 M LiBr at 25 °C with a flow rate of 0.5 mL/min. 3.0 mg of HCP polymer was dissolved in DMF solution (0.6 mL) containing LiBr (0.1M) and left to stand overnight. The polymer solutions were filtered with 0.45 μm PTFE filters before injecting into the SEC system. SEC analysis of the hydrophobe containing polypeptoids was performed by using an Agilent 1200 system equipped with three Phenomenex 5 μm, 300 × 7.8 mm^2^ columns, a Wyatt DAWN EOS multiangle light scattering (MALS) detector (GaAs 30 mW laser at λ = 690 nm), and a Wyatt OptilabrEX differential refractive index (DRI) detector. The data analysis was performed by using Wyatt Astra V 5.3 software. The PDI were obtained by using polystyrene standards.

### Liposome Preparation

The liposomes were prepared by the thin-film hydration technique where the lipids are first dissolved in an organic solvent and then evaporated to form a lipid thin film. Typically, 100 mg of PC lipid was dissolved in 15 mL of a chloroform and methanol mixture (2/1, v/v) in a round-bottom flask. The solvent was then evaporated on a rotavapor (Buchi R-205) at room temperature at 100 mbar for 3 h to form a thin lipid film. The film was further treated in a vacuum at 6 mbar for 30 min to remove residual solvent. The formed thin lipid film was then hydrated by using DI water at 50 °C, which yielded a suspension of large lipid vesicles. FITC–dextran loaded vesicles were prepared in a similar way with the exception of using FITC–dextran solution in DI water for the hydration step. The lipid film was hydrated by using 1 mg/mL of FITC–dextran solution at 50 °C for 30 min. The vesicle suspension was extruded 21 times through polycarbonate membrane with a pore size of 100 nm to downsize the unextruded vesicles into small unilamellar vesicles with an average diameter of 100 nm.

### Cryo-SEM

A Hitachi S-4800 field emission scanning electron microscope with the operating voltage of 3 kV was used to obtain cryogenic SEM images of emulsions and bacterial biofilm. Samples were loaded into rivets mounted onto the cryo-SEM sample holder. The samples were then plunged into slushed liquid nitrogen to freeze the sample. This was followed by fracturing at −130 °C using a flat-edge cold knife and sublimation of the solvent at −95 °C for 15 min to etch the sample. The temperature was lowered back to −130 °C, and the sample was then sputtered with a gold–palladium composite at 10 mA for 132 s before imaging.

### Cryo-TEM

The morphology of the complexes was characterized by a FEI Tecnai G2 F30 twin transmission electron microscope operated at 300 kV equipped with SDD EDS for elemental mapping. Cryo-TEM imaging was done on an FEI G2 F30 Tecnai TEM operated at 150 kV. To prepare the sample, a 200-mesh lacey carbon grid (Electron Microscopy Sciences) was picked up with tweezers and mounted on the plunging station of an FEI Vitrobot. Four microliters of the solution was applied to the grid. The excess liquid was blotted by filter paper attached to arms of the Vitrobot for 2 s to form a thin film. The sample was then vitrified by plunging into liquid ethane. The vitrified sample was finally transferred onto a single-tilt cryo specimen holder for imaging.

### Small-Angle X-ray Scattering (SAXS)

SAXS experiments were performed at the Advance Photon Source on beamline 12-BM. All measurements were conducted with the 12 keV beam at 25 °C. The samples were loaded in 1.5 mm quartz capillaries and placed on a sample holder at a sample-to-detector distance of 2 m. The data are presented as absolute intensity versus the wave vector *q* = 4π sin(θ/2)/λ, where λ is the wavelength and θ is the scattering angle. The reduction of SAXS data and background subtraction were performed by using Irena SAS macros on Igor Pro software.

### Fluorescent Microscopy

FITC–dextran was encapsulated within liposomes by hydrating lipid film with an aqueous solution of FITC–dextran. The loaded liposome suspension was transferred to a syringe and extruded 21 times through an 800 nm polycarbonate membrane. The reason for extrusion through 800 nm membranes is based on the fact that the larger vesicles show a clear specular pattern on the fluorescence micrographs. Unencapsulated FITC–dextran was removed by dialyzing through a dialysis bag (MW cutoff: 30 kDa) against a 100:1 deionized water bath volume at 25 °C for 1 h. Fluorescent microscopy images were taken with a Nikon A1 confocal microscope. A 20 μL sample was pipetted onto a standard microscope slide. A 488 nm laser was used to excite the fluorescence-tagged samples, and the emission wavelength was 525 nm.

## Results and Discussion

**Figure 3:**
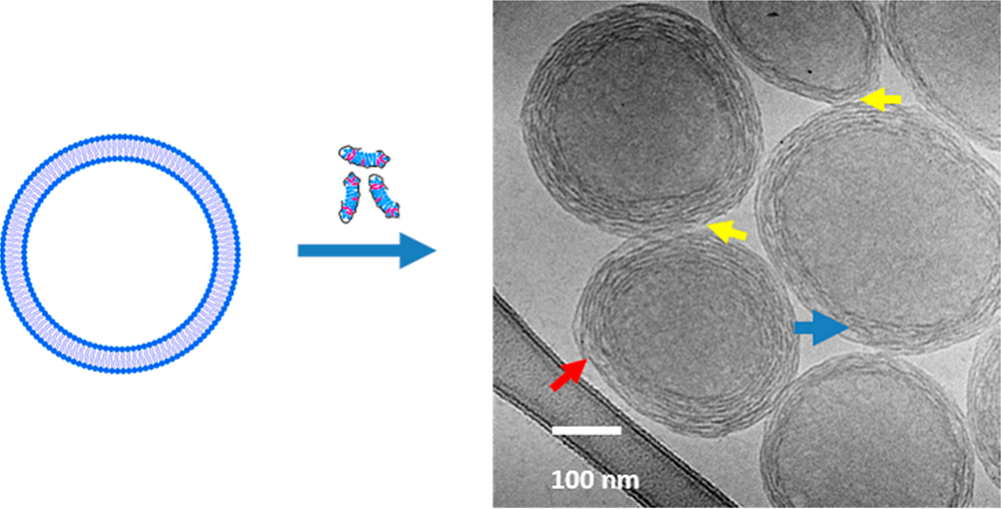
Multilayered vesicle formation: liposomal templates (0.25 wt % lipid) and HCP–lipid (0.25 wt % lipid and 0.5 wt*%* HCP) complexes are mixed to generate these multilayered vesicles (final concentrations: 0.25 wt % lipid, 0.17 wt % HCP). The waviness of the layers and the lack of full continuity (blue arrows) are shown. The yellow arrows point to potential connections between these vesicles, and the red arrow is a free ending of a layer.

Figure 3 illustrates the primary finding that we seek to understand in this paper. The concise description of the phenomenon is as follows: (1) upon mixing lipid vesicles (Figure 2) with HCP at a lipid-to-HCP weight ratio of 1:2 the vesicles rupture and equilibrate into ∼10 nm fragments (Figure 1b); (2) the fragments when mixed with fresh liposomes (2:1 volume ratio of liposomes (0.25 wt % lipid) to HCP–lipid complexes (0.25 wt % lipid, 0.5 wt % HCP) lead to the formation of a fascinating structure of multilayered vesicles as shown in Figure 3. On close examination of the vesicles with cryo-TEM, we observe the following: (1) the layered vesicles are usually larger than the original PC liposomes, (2) the layers do not appear to be continuous, and (3) the individual layers appear to be more loose and flexible rather than the tight curvatures formed in liposomes or traditional multilamellar vesicles (MLVs). Because the layers do not appear to be continuous lamelli, we do not refer to these structures as multilamellar vesicles but rather as multilayered vesicles. We also see what appears to be connections between these structures, but it is not entirely clear if these are actual connections or if these result from small overlaps between adjacent vesicles.

**Figure 4:**
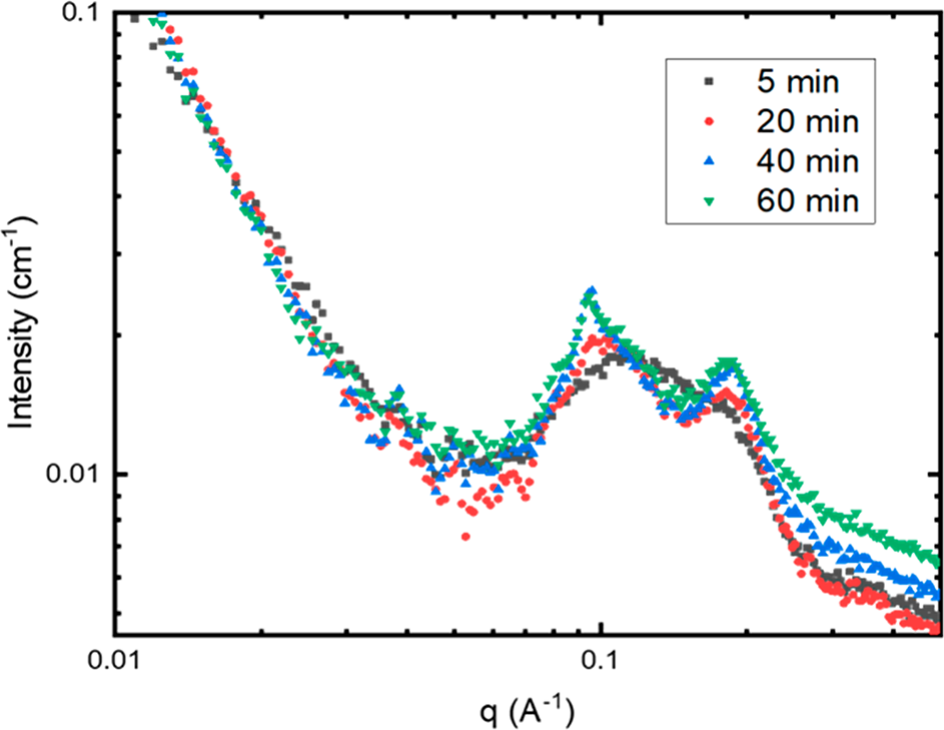
Transition to multilayered vesicles upon HCP–lipid complex addition from 1 to 60 min analyzed by SAXS. Diffraction peaks begin appearing at 40 min postmixing, indicating the presence of multilayered vesicles.

We conducted a time-dependent SAXS analysis of the process of the multilayer formation at the Advanced Light Source at Argonne National Laboratory, with the results shown in Figure 4.

The sample was kept stationary in a capillary, and the high flux of the synchrotron X-ray radiation allows sufficient data acquisition in 10 s, providing an opportunity to capture the scattering curves as the sample undergoes transitions in real time. The *q*^–2^ decay at low *q* is indicative of the presence of bilayer structures.^28^ Broad diffraction peaks emerge as early as 20 min and sharpen and stabilize around 40 min. As the incubation time increases, the signal reveals the diffraction peaks at *q* = 0.095 A^–1^ and *q* = 0.19 A^–1^, where the first peak indicates a repeat distance *d* of 6.6 nm and the second peak is the higher-order peak verifying a lamellar structure. The broadness of the peaks is perhaps correlated to the fact that the these are multilayered structures with wavy sheets and with rather imprecise spacings.

**Figure 5:**
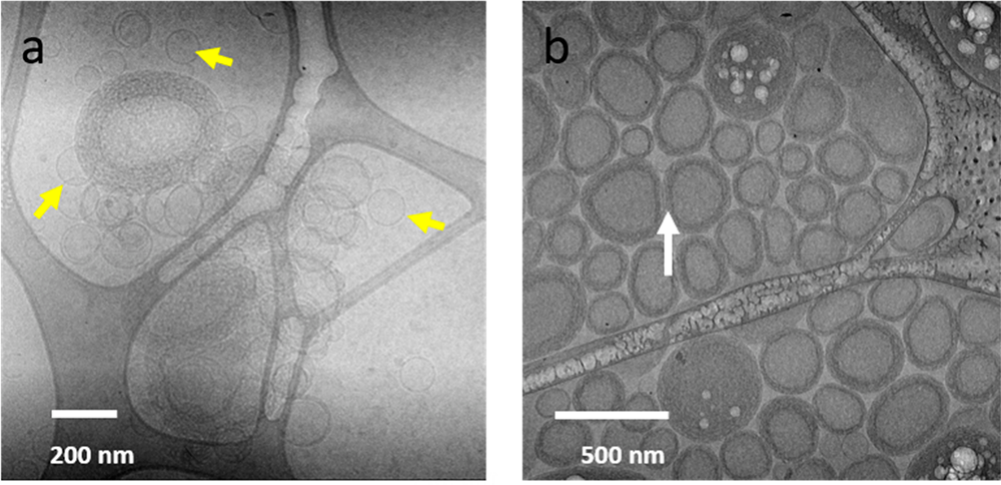
Structural transitions as a function of time: (a) aggregation of vesicles is observed 20 min postmixing of complexes and liposomes together with remaining unilamellar vesicles are present (yellow arrows); (b) multilayer vesicles are formed in 4 h postmixing. The flexibility of the layers in conforming curvature to adjacent vesicles is noted by the white arrow. The system consists of (0.5% HCP25 + 0.25% lipid) that form the HCP–lipid complexes added to 0.25% lipid containing vesicles to obtain a final concentration of 0.17% HCP25 and 0.25% lipid.

We have also examined the evolution of the multilayered structure through cryo-TEM as shown in Figure 5.

Vitrifying the sample after 20 min shows a transition from the essentially unilamellar structures of Figure 5 (left) to a system containing a mixture of remnant unilamellar liposomes with the emergence of bilayered and multilayered vesicles. It is also important to note the observation of vesicle clustering. The clustering could be the initial step of multilayered vesicle formation where the HCP–lipid complexes attach to unilamellar vesicles and bring vesicles together. It is also possible, although somewhat speculative, that the depletion effect of adding small colloids (the HCP–lipid complexes) to the much larger liposomes leads to the clustering of the larger liposomes following which bridging and growth into multilayered structures occur. We note, however, that we have not seen the growth into layered structures with polypeptoids where all the nitrogen substituents are the methoxyethyl moieties (Figure 1), clearly indicating that it is the alkyl hydrophobes on the backbone responsible for this transition.^11^

When the sample is incubated at room temperature for 4 h, large areas of the grid contain the multilayered vesicles as shown in Figure 5 on the right. We see a flexibility in the curvature of the multilayered vesicles and a tendency for two adjacent vesicles to flatten. In the 4 h time period, we also see some extremely large multilayered vesicles as shown in the Supporting Information S1 which also illustrates additional cryo-TEMs of the flexible vesicles. Again, we note the novelty of this transition from unilamellar liposomes to multilayered vesicles through the addition of such HCP–lipid fragments. The literature cites several examples of polymers being able to break liposomes into fragments, and this is indeed the basis for membrane protein extraction through the use of styrene–maleic acid (SMA) amphiphilic polymers (amphipols)^29−31^ and dendrimers.^32^ However, we are unaware of any studies where such polymer–lipid fragments have been reported to bridge bilayers to form multilayered vesicular structures.

**Figure 6:**
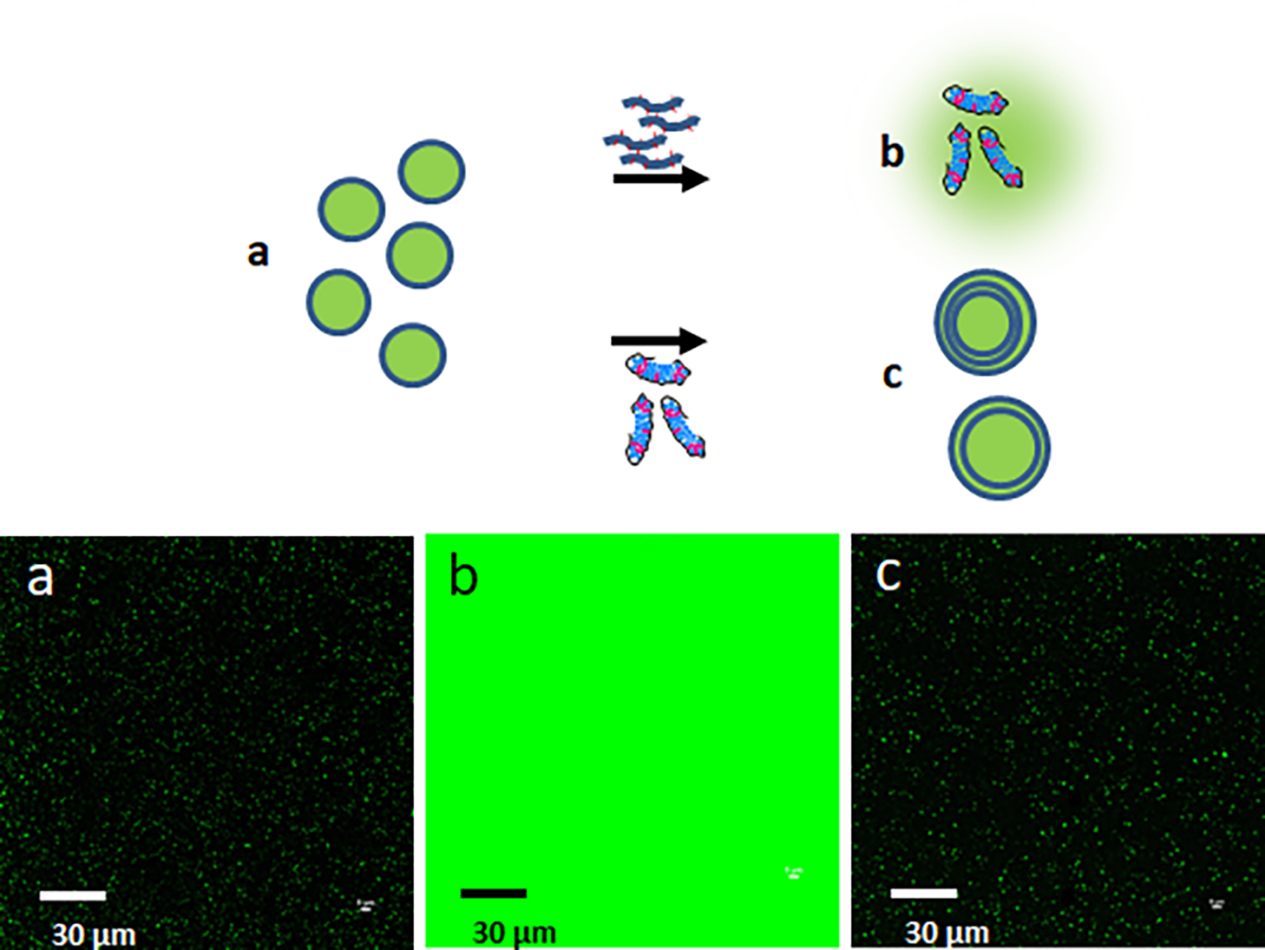
FITC–dextran leak test to show retention of liposomal cargo upon transformation from unilamellar to multilayer vesicles. (a) represents 0.25 wt % liposomes loaded with 0.02 wt % FITC–dextran (4 kDa). (b) represents the system when 0.5 wt % HCP is added to disrupt the liposomes and release the dye into solution. (c) represents the case where the HCP–lipid complexes (final lipid concentration 0.25 wt % and HCP 0.17 wt %) is added to system (a) to generate multilayer vesicles without significant release of the dye. The circle represents a liposome bilayer. We note that this experiment was conducted with 800 nm vesicles to clearly visualize the fluorescent specular pattern in the micrographs.

A crucial part of understanding the mechanism of multilayer formation is in assessing the structural stability of the added lipid vesicles acting as templates for growth of the layers. In other words, it is necessary to understand if liposomes originally loaded with a water-soluble drug lose their cargo when they transition to the multilayer structure. Accordingly, we loaded liposomes with fluorescent FITC–dextran and then added the HCP complex to this system as shown in the pathway from a to b in Figure 6.

The HCP complexly disrupts the liposomes releasing the fluorescent dye as seen in the transition from the bright pinprick-type fluorescent pattern in system a to the broad background fluorescence in system b. The pathway from a to c is one where liposomes loaded with FITC–dextran are contacted with the HCP–lipid complexes. The retention of the bright dot pattern in system c is an indication that there is negligible dye leakage in this pathway. Thus, the observation indicates that there must be clear fusion or bridging between liposomes in the creation of the multilayers to allow retention of the cargo in the multilayered structure.

**Figure 7:**
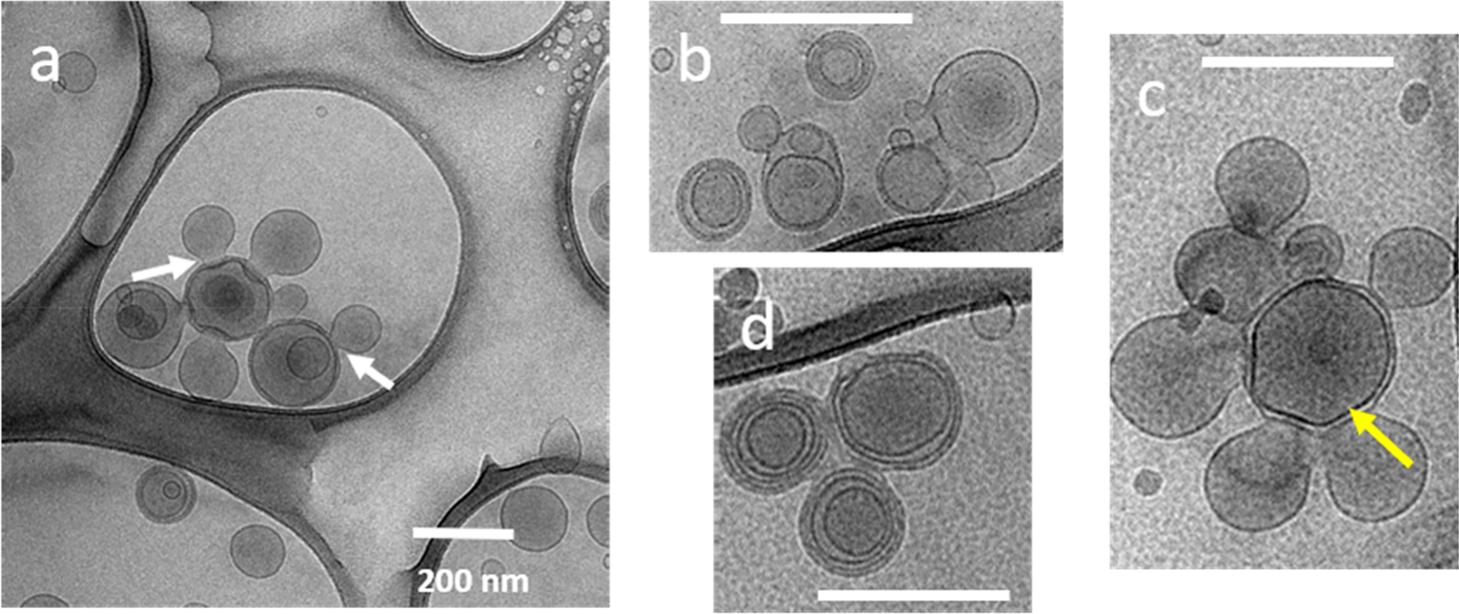
Vesicle clustering and fusion as an initial step, upon addition of a small quantity of the HCP–lipid complexes. 10 vol % of HCP–lipid complexes (relative to liposomes) are added to fresh liposomes to understand the transition to multilayered vesicles (0.25 wt % lipid and 0.05 wt % HCP final composition, 24 h incubation). (a) Five outer vesicles attempting to fuse into the center vesicle results in a clustered state where fusion necks are observed (white arrow). (b), (c), and (d) are additional images at higher magnifications that show the clustering and the flattening of inner layers (yellow arrow) that we attribute to the incompleteness of the layers and internal pressure gradients from the fusion process. All scale bars are 200 nm.

To try to arrest the formation of the multilayered liposomes, we conducted an experiment where we added just a small aliquot of the HCP–lipid complexes (10 vol % of the level used to rapidly form the multilayered vesicles) to fresh liposomes and incubated the system for 24 h prior to vitrification and imaging. Interestingly, as Figure 7 illustrates, there is clear evidence of vesicle fusion that is arrested. We have taken images from various parts of the TEM grid to show regions of multiple fused vesicles, some containing multiple layers.

We also observe fusion “necks” where bilayers join (yellow arrows), and in vesicles with just a couple of layers we often see a flattening of layers (blue arrows), again indicating flexibility in the layers that may be made up of bilayer strands rather than a complete bilayer. In a sense these cryo-TEM images provide a rationale for the fact that large molecule contents of vesicles do not leak out during fusion which may be the initial step to multilayer vesicle formation. The observation is very similar to the vesicle fusion that is done by SNARE proteins (snap receptor proteins) that mediate neurotransmitter release,^33,34^ although we note that the literature on SNARE proteins does not address the formation of multilayered vesicles. Thus, we see HCP–lipid complexes as being able to mimic SNARE protein behavior by being fusogenic to vesicles and at high concentrations being able to form multilayered vesicles. The literature cites other examples of systems that induce fusion. In a fascinating example, carbon nanotubes have been shown to induce vesicle fusion through insertion of the nanotube into the bilayers of adjacent vesicles and allowing a sliding of lipid molecules along the hydrophobic surface of the nanotubes.^35^ Metal ion binding to amphiphilic ligands consisting of synthetic bipyridine lipoligands has been reported to induce fusion of vesicles leading to giant vesicles.^36^ These results indicate that bridging vesicles could be a general phenomenon to induce fusogenesis. We note that addition of the HCP–lipid complexes to 40% of the level required to rapidly form the multilayered vesicles also leads to multilayered vesicles albeit seemingly with a reduced number of layers. The results are shown in Supporting Information S2 and perhaps point to variations in the rate of formation of the multilayered vesicles as a function of the concentration of the complexes. We also note the flexibility of the curvatures of the multilayered vesicles shown in Supporting Information S2.

**Figure 8:**
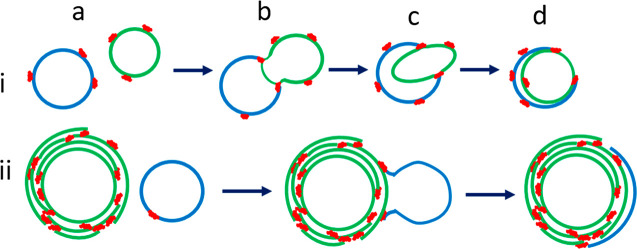
Potential transition mechanisms of unilamellar vesicles to multilayered vesicles: The top row (i) shows the process of fusion of two unilamellar vesicles (a) to a two-vesicle cluster (b) followed by engulfment (c) and the formation of a bilayered vesicle (d). The bottom row (ii) shows the continuation of the process to multilayered vesicles with layers that are not continuous. The colors blue and green represent separate vesicles, and the color red represents HCP–lipid complexes attached onto the vesicles.

On the basis of these observations, we propose a mechanistic model of multilayered vesicle formation as shown in Figure 8.

First, it is recognized that the hydrophobic interaction is responsible for the tendency of the alkyl chains of HCP to shield themselves from water and embed into the lipid bilayer. Functionalization of the polypeptoid yields randomly distributed alkyl chains throughout the backbone. The HCP–lipid fragments are prepared from mixing lipid and HCP at a 9:1 molar ratio (0.25 wt % lipid with *M*_w_ 775 g/mol and 0.5 wt % HCP25 with *M*_w_ 13900 g/mol). Every molecule of HCP contains on average 25 randomly distributed decyl groups as the hydrophobes. If there are nine lipid molecules attached to each HCP, the number is translated to ∼1 lipid for every 3 hydrophobes. The observation that HCP on its own disrupts lipid bilayers implies that the 25 hydrophobes on each HCP molecule are available to insert into lipid bilayers and disrupt the bilayers. On the other hand, in the HCP–lipid complex, some of the hydrophobes are noncovalently attached to lipid species, and there are fewer hydrophobes available to create membrane disruption. We therefore propose that the HCP–lipid complexes attach to liposomes without disrupting them as a first step as shown in (a) of Figure 8.

Interestingly, when these complexes are added to fresh liposomes, some of the dynamically uncomplexed hydrophobes (the *n*-decyl groups) may bridge liposomes. The self-assembly to multilayered vesicles may begin with vesicle clustering through the depletion interactions brought about by the HCP–lipid complexes that are initially in solution prior to interaction with the vesicles. After attachment to a vesicle some of the nonintercalated hydrophobes then insert into lipid bilayers of an adjacent vesicle forming the fusogenic cluster as shown in (b) of Figure 8, which also indicates the formation of bridging necks of two vesicles undergoing fusion. (c) and (d) show the process of an outer vesicle (in green) engulfing an inner vesicle (in blue) to form a bilayered system. The sequence in the second row essentially shows addition of layers where a vesicle (blue) fuses to a layered vesicle (green) and then opens up to engulf the larger layered vesicle and thus add a portion of an additional layer. Stopping the process with insufficient HCP–lipid complexes that serve as a limiting reagent may lead to the formation of multilayered vesicles with attached single layer vesicles that are unable to fully fuse into another layer as shown in the cryo-electron micrographs of Figure 7. A somewhat similar transition has been proposed to understand the fusion when anionic DNA is attached to cationic vesicles and therefore bridges between vesicles.^37^ The mechanism of such induced fusion is electrostatics, while this work describes the bridging of vesicles by using the hydrophobic interaction. A fascinating glimpse of such hydrophobic interactions inducing bilamellar vesicle formation has been shown by Raghavan’s group using vesicles of a mixture of cationic and anionic surfactants and a hydrophobically modified chitosan, a 200 kDa cationic biopolymer.^38^ These researchers have clearly pointed out the role of the hydrophobically modified chitosan in bridging liposome layers in the final conformation of a mixture of unilamellar and bilamellar vesicles.

Thus, as shown in the second row (ii) of Figure 8, we hypothesize the layering of vesicles occurs by building around existing layers. A new layer cannot assemble in between existing layers but can only become the outermost one. The multilayered vesicle formation schematic in Figure 8 is an attempt to explain the existence of open bilayers wrapped around vesicles through HCP–lipid complex bridging. The resulting structures can possibly undergo transitions described in Figure 8 until all the HCP–lipid fragments are used. The mechanisms outlined in Figure 8 serve to describe the processes occurring during the transition in a sequence but potentially take place very quickly, leading to the stable conformation of multilayers as shown in Figure 3.

## Conclusions

To summarize the key aspects of this work, the addition of HCP–lipid complexes to lipid vesicles initiates a transformation to multilayered vesicles through an initial clustering and an engulfment of vesicles. It is remarkable that the clustering and engulfment retain large molecule intravesicular cargo without spillage into the bulk aqueous medium. The process can be controlled through the amount of the complexes that are added, leading to an arresting of intermediate structures en route to the formation of multilayered vesicles. The layers of the multilayered vesicles appear to be made of incomplete sheets of lipid bilayers which may be connected or bridged by the HCP–lipid complexes. The entire process is a consequence of hydrophobe insertion into lipid bilayers through the hydrophobic effect.

The consequences of these observations are significant. These materials are protein mimics and may therefore be intrinsically biocompatible. However, the use of the term biocompatible requires specificity of the physiological environment as discussed by Williams,^39^ and we are cautious in using the term here. While our earlier work has shown that the HCP–lipid complex can enter cells,^26^ the observations shown here indicate that the complexes could be used in targeting intracellular organelles. The possibility of introducing several types of therapeutics into the lipid bilayers implies the use of these systems as potential multiple agent delivery vehicles. An additional advantage of a multilayered vesicle system is the possibility of reducing the permeability of the cargo in the aqueous core, thus allowing control of release profiles. There are other potential advantages. Such large vesicles could be designed to stay at the site of injection rather than be carried into the bloodstream. They may therefore have implications to drug delivery to tumor vasculatures. Degradation by phospholipases and ingestion by macrophages could also be reduced by such multilayered vesicle morphologies. These are areas of continued research.

## Supporting Information Available

Section S1: a cryo-TEM of a 4 h sample; section S2: cryo-TEM images of multilayered vesicles formed with a smaller aliquot of HCP–lipid complexes (PDF)
