# Supplementary material for: Hydrophobe Containing Polypeptoids Complex with Lipids and Induce Fusogenesis of Lipid Vesicles
Source: J Phys Chem B. 2021 Mar 17;125(12):3145–52. doi: 10.1021/acs.jpcb.0c11477 (PMC8041297; doi:10.1021/acs.jpcb.0c11477)
Supplement: Supplementary file 1 — jp0c11477_si_001.pdf [file jp0c11477_si_001.pdf]

Hydrophobe containing Polypeptoids Complex with Lipids and Induce Fusogenesis of Lipid Vesicles

Marzhana Omarova[1], Yueheng Zhang[1], Igor Kevin Mkam Tsengam[2], Jibao He[3], Tianyi Yu[2], Donghui Zhang[2*] and Vijay John[1*]

1. Department of Chemical and Biomolecular Engineering

   Tulane University, New Orleans, LA 70118, United States

2. Department of Chemistry

   Louisiana State University, Baton Rouge, LA 70803, United States

3. Coordinated Instrumentation Facility

   Tulane University, New Orleans, LA 70118, United States

Supporting information

Number of figures: 2

Figure S1. Structures other than spherical multilayered vesicles are present in the sample 4 h after mixing of {0.5% HMP25, 0.25% PC liposomes} complexes + 0.25% PC liposomes, final conc. 0.17% HMP25, 0.25% PC liposomes.

Figure S2. Large multilayered vesicles are present in the samples with complexes and liposomes. 40v% of the lipid-HMP complexes is added to liposomes, final concentration 0.25wt% lipid, 0.14wt% HMP: (a) multilayered lipid membrane bends as vesicles aggregate, (b) multilayered vesicles with diameters over 300 nm are present in the samples.

*Corresponding authors. Vijay T. John, phone: 504-865-5883 e-mail: vj@tulane.edu. Donghui Zhang, phone: 225-578-4893 email: dhzhang@lsu.edu

Supporting Information

S1. Time dependent cryo-TEM images were obtained by vitrification of the samples at time points before complete equilibration. At 40 min post mixing of lipid-HMP complexes and liposomes the multilayer formation had already proceeded to the extent that no unilamellar vesicles are observed. Below is an additional image captured at 40 min post complex and liposome mixing. The image shows the presence of structures different from intact spherical miltilayered vesicles. The multilayer nature is still present, although the vesicle shape is distorted.

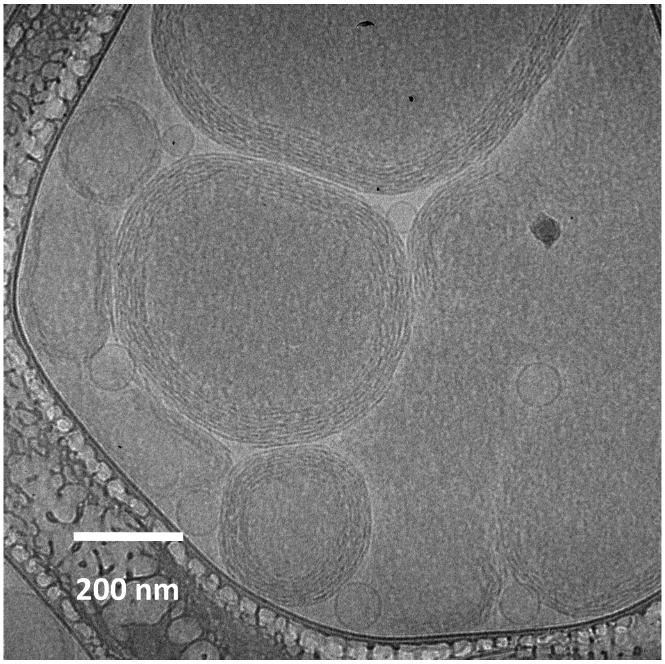

Figure S1. Structures other than spherical multilayered vesicles are present in the sample 4 h after mixing of {0.5% HMP25, 0.25% PC liposomes} complexes + 0.25% PC liposomes, final conc. 0.17% HMP25, 0.25% PC liposomes.

S2. Formation of multilayered vesicles discussed in the main text occurs through mixing of lipid-HMP complexes and liposomes at a 1:2 ratio, thus the volume of complexes is 50% of the volume of liposomes before mixing. By adding the complexes at a concentration less than 50% we attempt to gain more insight into the multlayered vesicle formation. Images below demonstrate that when complexes are added at the concentration of 40v% fewer layers are formed around liposomes. Additionally, large vesicle distortion and bending of the vesicle membrane is observed.

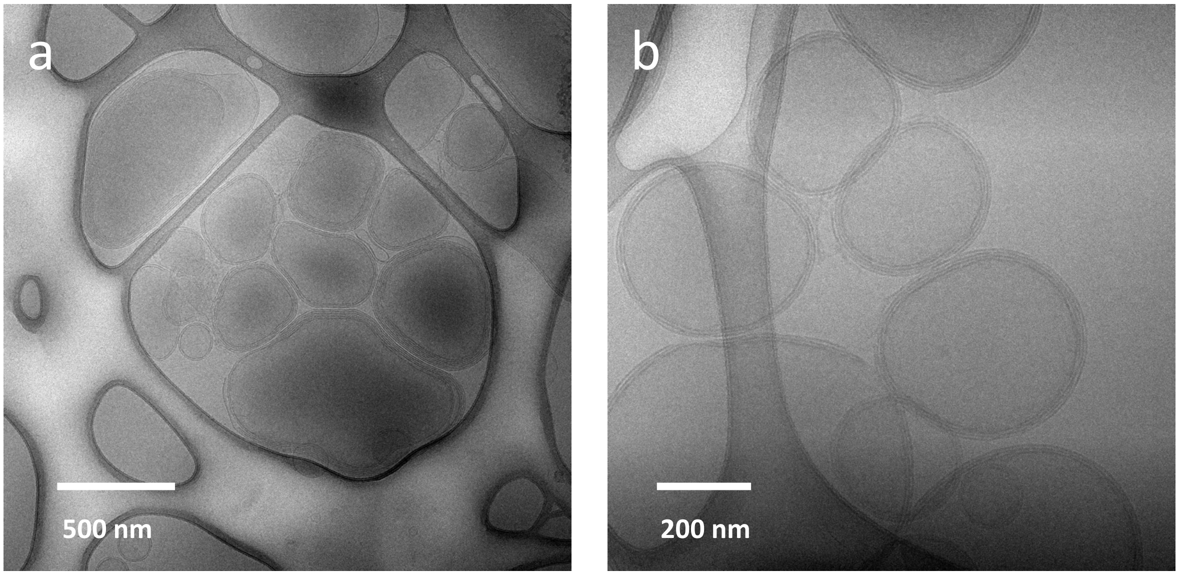

Figure S2. Large multilayered vesicles are present in the samples with complexes and liposomes. 40v% of the lipid-HMP complexes is added to liposomes, final concentration 0.25wt% lipid, 0.14wt% HMP: (a) multilayered lipid membrane bends as vesicles aggregate, (b) multilayered vesicles with diameters over 300 nm are present in the samples.
